# A robust prediction model for evaluation of plastic limit based on sieve # 200 passing material using gene expression programming

**DOI:** 10.1371/journal.pone.0275524

**Published:** 2022-10-03

**Authors:** Muhammad Naqeeb Nawaz, Sana Ullah Qamar, Badee Alshameri, Muhammad Muneeb Nawaz, Waqas Hassan, Tariq Ahmed Awan

**Affiliations:** 1 National University of Sciences and Technology, Islamabad, Pakistan; 2 National University of Technology (NUTECH), Islamabad, Pakistan; University of Sulaimani, IRAQ

## Abstract

This study aims to propose a novel and high-accuracy prediction model of plastic limit (PL) based on soil particles passing through sieve # 200 (0.075 mm) using gene expression programming (GEP). PL is used for the classification of fine-grained soils which are particles passing from sieve # 200. However, it is conventionally evaluated using sieve # 40 passing material. According to literature, PL should be determined using sieve # 200 passing material. Although, PL_200_ is considered the accurate representation of plasticity of soil, its’ determination in laboratory is time consuming and difficult task. Additionally, it is influenced by clay and silt content along with sand particles. Thus, artificial intelligence-based techniques are considered viable solution to propose the prediction model which can incorporate multiple influencing parameters. In this regard, the laboratory experimental data was utilized to develop prediction model for PL_200_ using gene expression programming considering sand, clay, silt and PL using sieve 40 material (PL_40_) as input parameters. The prediction model was validated through multiple statistical checks such as correlation coefficient (R^2^), root mean square error (RMSE), mean absolute error (MAE) and relatively squared error (RSE). The sensitivity and parametric studies were also performed to further justify the accuracy and reliability of the proposed model. The results show that the model meets all of the criteria and can be used in the field.

## Introduction

The plastic limit (PL) can be defined as the water content at which soil changes from plastic to semi-solid state [1–4]. It is often used to measure the physical and mechanical responses of soils and is regarded as a critical parameter in the development and design of geo-structures [5–8]. The most basic application of the plastic limit is to categorize fine-grained soils and their co-relation with nearly all mechanical properties of cohesive soils such as compressive strength, shear strength, toughness index, consolidation behavior, shrinkage and swelling characteristics, activity, stress history etc. [3,9]. Plasticity index (PI) is regarded as an index to distinguishes a problematic soil from a good quality soil, because soils with greater PI values are considered troublesome and undesirable for the most of construction projects.

Plastic limit (PL) is commonly determined in laboratory in accordance with ASTM-D4318 [10] and BS-1377-2 [11]. PL is used to categorize fine-grained soils, which are soils with particle sizes smaller than 0.005 mm according to ASTM standards [12]. Instead, it is evaluated based on material passing through sieve # 40 (0.425 mm particles) in accordance with ASTM-D4318 [10]. The problem is whether determining PL using sieve # 40 passing material is appropriate because it may contain coarse grains particles i.e., sand. The effect of coarse content in clayey soils has been discussed in literature. This results in significant changes in soil classification and subsequent correlations of PL with mechanical properties of soils.

Several studies have been done in the literature to address the issue, which is that the PL must be evaluated using material passing through sieve # 200 rather than material passing through sieve # 40. Polidori [13] proposed a modified plasticity chart based on Atterberg’s limits determined with particle sizes smaller than 0.075 mm. This study proposed significant changes in Casagrande’s plasticity chart and indicated differences in silt and clay zones based on soil classification utilizing sieve # 200 soil material. It was observed that the presence of coarse grained soil influences the Atterberg limits and causes variations in the Casagrande chart.

Polidori [14] also investigated subsequent variations in PL dependent on particle size by illustrating the link between clay content and Atterberg limits. It was observed that clay content has an influence on Atterberg limits and exhibits a linear increasing trend as Atterberg limits increase. Polidori [15] also introduced a novel soil classification technique for two different soil types.; (1) inert; (2) active binder. It has been observed that clay content, particularly clay minerals, has a considerable impact on plasticity, leading to changes in the USCS.

Moreno Moroto et al. [16] presented a critical review of various soil classification systems, highlighting fundamental limitations of multiple classification systems, including the USCS and the Polidori plasticity chart. According to this study, the Moreno-Moroto soil classification system has better predictive ability than other systems because of its distinct selection criteria, simplicity, accuracy, and adaptability to demands. Further, Lekan et al. [17] compared the Atterberg limits of laterite soil using material passing from sieve # 40 and #200 and reported significant changes in Atterberg limits values based on two different methods. This validates the concept that determining plastic limit using sieve # 40 material may result in erroneous assessments because sieve # 40 material passing may include a considerable number of coarse particles, which have an inverse relation with plastic limit.

According to ASTM-D4318 [10], when using plasticity tests to assess the properties of a soil, the relative contribution of this portion of the soil to the properties of the sample as a whole must be properly considered. This is because the plasticity tests are only conducted on that portion of a soil that passes 425 μm (No. 40 sieve). Nagraj et al. [18] proposed the study in which the plastic limit has been used as a correlation parameter to assess the compaction characteristics of natural soil as a whole, and it has been changed to account for the percentage of soil fraction less than 425 μm present in the soil. But even so, none of the previous studies considered this method of accounting for the amount of fines less than 425 μm present in the soil when establishing the correlation equations [19]. Hence, this study utilizes the concept of determination of PL considering particle having size less than 0.075 mm based on the previous advancements in understanding the plasticity behavior.

However, it is certain that determination of PL, particularly PL_200_ is arduous, tedious and challenging task that generally needs multiple attempts to obtain correct results. In this case, artificial intelligence (AI) based prediction models are considered useful due to the effectiveness in terms of cost and time, and capability to incorporate multiple influencing parameters [20,21].

Various research efforts have been made in recent years to determine Atterberg limits indirectly using conventional data science methodologies. For instance, Seybold et al. [22] used multiple linear regression (MLR) to develop a prediction model for estimating Atterberg limits depending on clay content (C) and cation exchange capacity (CEC) as input parameters. According to this study, the C and CEC are critical in determining Atterberg limits. Keller & Dexter [23] proposed correlation of Atterberg limits and clay content. These studies were dependent on plastic limit determination using sieve # 40 passing material and did not take into account the plastic limit determination using sieve # 200 passing material. Moreover, it has actually been recognized that PL of soil is dependent on clay, silt, and coarse content [24]. The earlier studies have used an experimental route to determine PL using sieve # 200, and no attempt has been made in the recent times, to the best of the authors’ knowledge, to predict PL_200_ using gene expression programming (GEP) that integrates clay, silt, and sand content.

The goal of this research is to propose, a novel prediction model of PL_200_ based on experimental data collected from laboratory testing. Soil samples were collected from multiple locations in Islamabad, Pakistan, and experimentally tested to determine the plastic limit as well as basic index properties of soils such as clay content (CL), silt content (ML) and sand content (S). Moreover, a PL_200_ prediction model was developed utilizing the GEP machine learning approach. Various statistical tests and error plots were used to validate the suggested prediction model. Subsequently, parametric and sensitivity tests were also done to support the prediction model.

### Basics of Genetic Programming (GP) and Gene Expression Programming (GEP)

**Fig 1:**
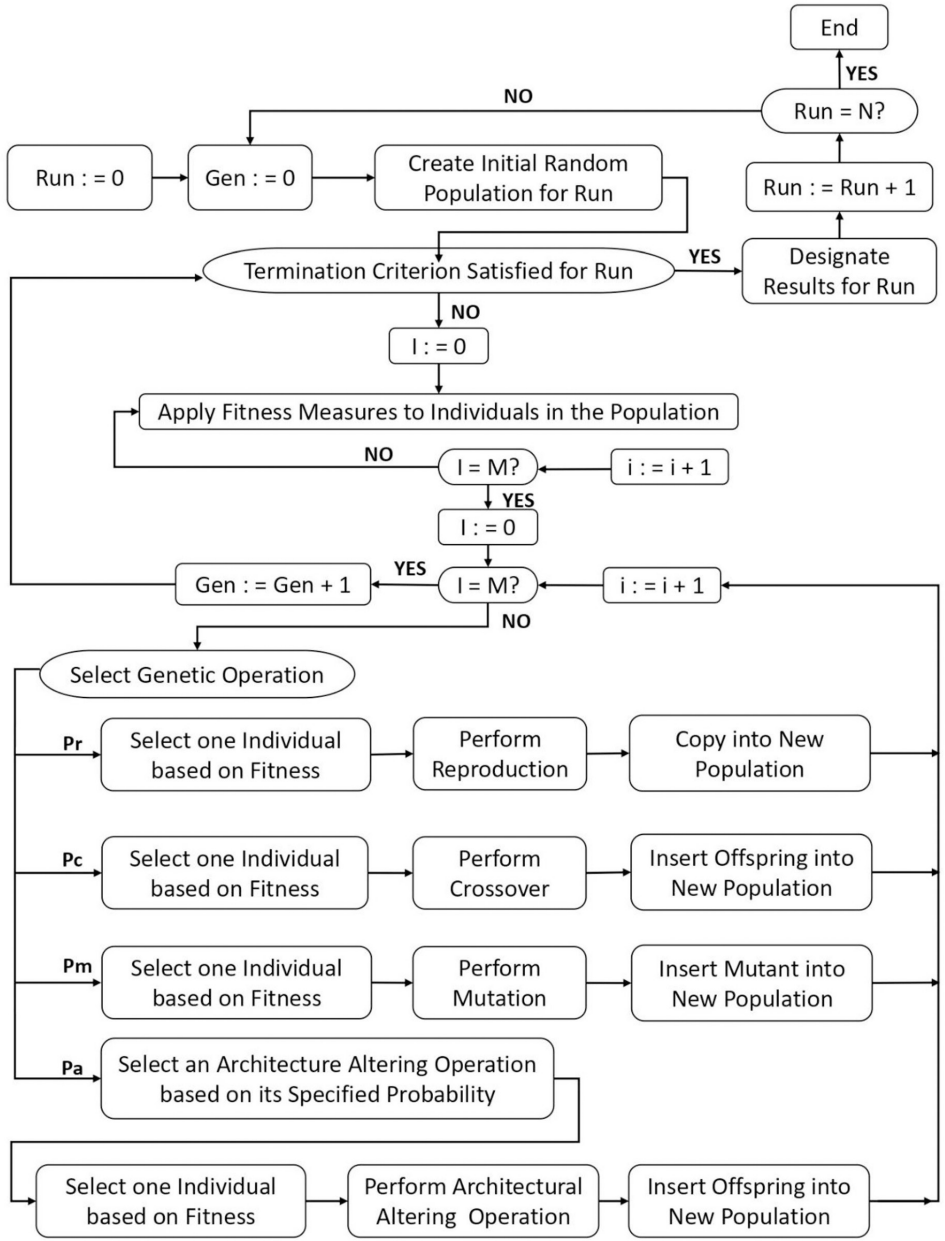
Process of Genetic Programming (GP).

**Fig 2:**
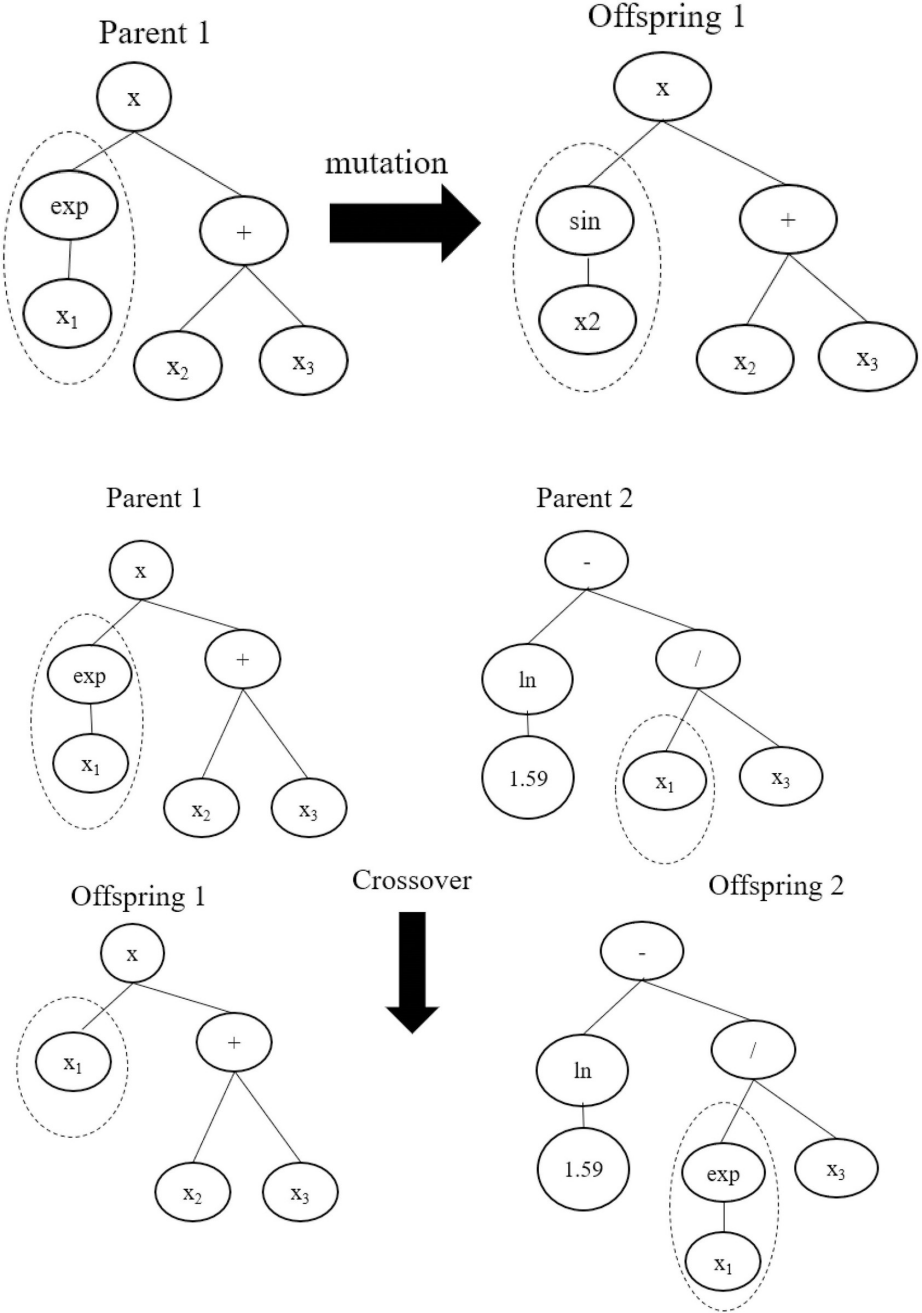
General procedure of mutation and crossover.

Genetic algorithm (GA) is a stochastic method which uses principles of genetics for finding the optimal solution of a problem. Genetic programming (GP) is an improved form of GA and was introduced by Koza and Poli, [25]; Nazari and Torgal, [26]. In GP, a computer program is evolved to solve the problems based on the evolutionary biological mechanisms such as mutation, cross over and reproduction [27]. The mutation is a biological evolutionary process in which a new offspring (solution) is produced by flipping a part of string or gene whereas in crossover, solution is created by swapping string or genes from two parents [28]. The working principles of GP along with mutation and crossover have been demonstrated through Figs 1 and 2.

Gene expression programming (GEP) is the modified form of GP and is widely appreciated by the researchers in the field of civil engineering [29–34]. For instance, Jalal et al, [35] developed prediction models for the assessment of compaction characteristics of expansive soils using GEP. Armaghani et al, [36] deployed GEP to propose the prediction model of uniaxial compressive strength of soils. Mousavi et al, [20] proposed GP based correlation models to predict shear strength of soil. The main advantage of using GEP is that it provides robust mathematical relations which are more beneficial for engineers working in the field. Therefore, various studies have incorporated the application of artificial intelligence (AI) based techniques to devise more sustainable, cost effective and less time-consuming solutions in the field of geotechnical engineering [26,37–44].

In GEP, the parameters / chromosomes are linked in the form of expression trees (ETs) which tend to adapt and learn by varying their sizes and shapes which are initially encoded as fixed size linear strings (genome). A multi-genic chromosome is further divided into number of genes and each Sub-ET consists of head and tail. These are the places where genetic operators are deployed to produce new solutions. In GEP, genetic operator is used to develop empirical correlations by combining different influencing input parameters and arithmetic functions (+, -. *, ÷, sine, cosine, tan etc.,). The arithmetic functions and constants are referred to as function set and terminal sets respectively. Karwa language is used to infer the data and information stored in a chromosome to further process the formulation of mathematical expression from ETs [45]. The principle of deducing equation using Karva language is to simply read the expression tree (ET) generated by GEP, from left to right and from top to bottom (same as we read a text page).

**Fig 3:**
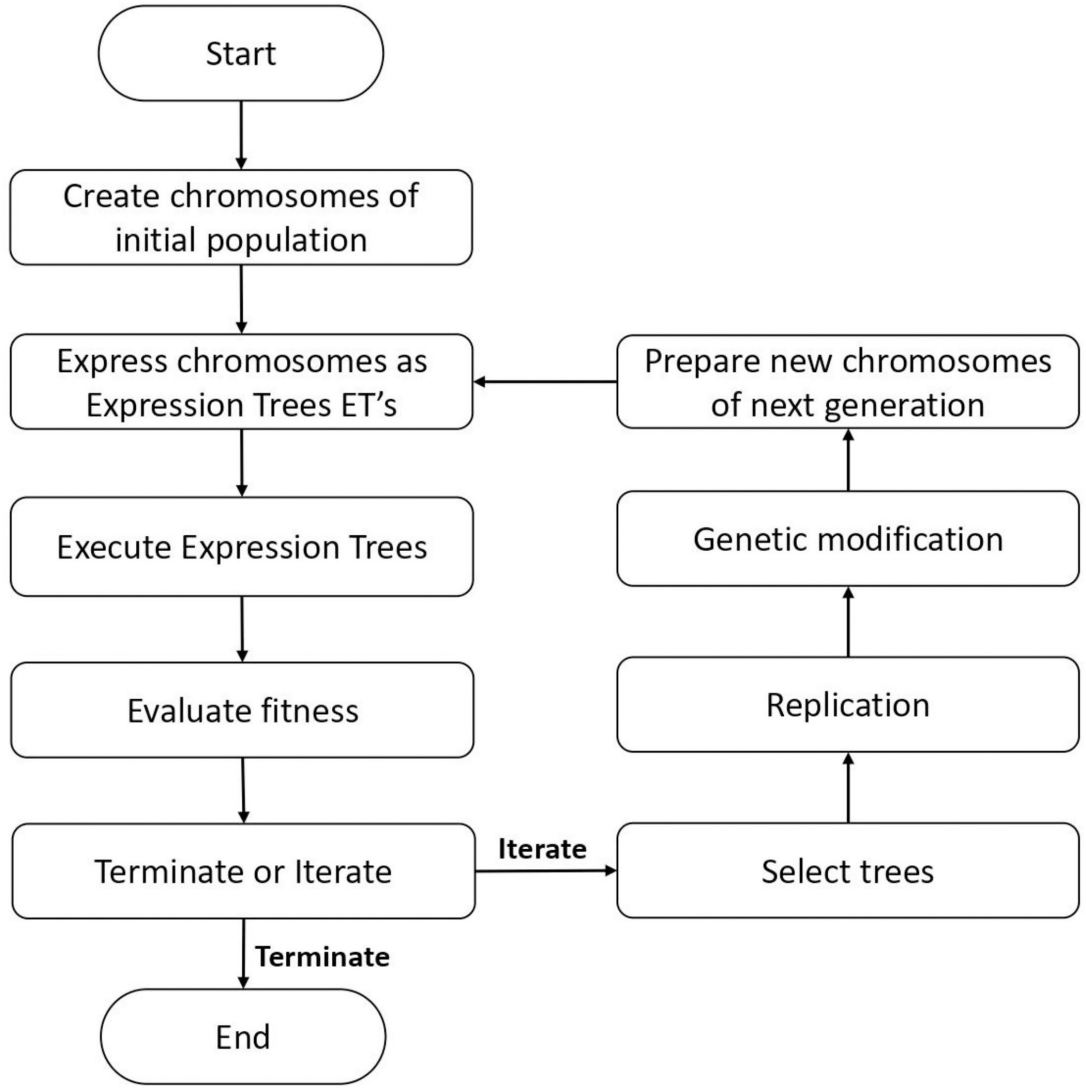
Steps involved in developing algorithm of GEP.

The flowchart in Fig 3 shows the working principles of GEP. The process initiates with the generation of initial random population in accordance with terminal setting and function, for all the individuals. Then chromosomes are expressed in the shape of expression trees (ETs), and afterwards a best fit solution upon evaluation of fitness is processed for the next generation. The fitness of chromosome can be evaluated using various statistical checks and the notable examples are means absolute error (MAE), root mean square error (RMSE), relative standard error (RSE) and correlation co-efficient (R^2^). The iterative procedure is continued until the desired solution is achieved. Conversely, Roulette wheel method is deployed to select best fit solution of first iteration and then new population of chromosomes is created by the process of mutation, cross over and reproduction. This process of iterations is stopped when best threshold criteria of selection is obtained.

## Materials and methods

### Geological database

The soil samples were collected from different locations of Pakistan (Islamabad, Khyber Pakhtunkhwa and Punjab). The samples were retrieved from shallow depths ranges between 1 to 2 m. The geology of Islamabad area comprises silty clayey and clayey silt type of soils along with siltstone, gravels, sandstone, shale etc. at varying depths. The laboratory testing program was formulated to obtain primary index characteristics of soils such as sand content (S), clay content (C), silt content (M) and plastic limit using sieve # 40 (PL_40_) and 200 (PL_200_) passing materials.

### Experimental methods

The sieve analysis test was performed in accordance with ASTM D 422 to determine percent sand and fine material [46]. In this test, oven-dried soils are passed through a series of sieves ranging from # 4 (4.75 mm) to # 200 (0.075 mm) in descending order. The percentage of material which passes from sieve # 200 is categorized as fine material whereas material retained on sieve # 200 and passing from sieve # 4 (4.75 mm opening size) is referred as sand content (S). The fine content is further sub divided into clay (C) and silt (M) content.

The C and M are types of soils which are comprised of particles smaller than 0.075 mm size and are therefore cannot be determined using sieve analysis method. Therefore, hydrometer analysis of particle sedimentation is commonly used for the determination of C and M [47]. This test is performed by mixing soil particles with size less than 0.075 mm with water and dispersing agent (sodium hexameta phosphate or sodium silicate) to neutralize the soil particles to prevent reaction of clay particles and water. Afterwards the relative movement of soil particles in suspension with regards to hydrometer device is recorded and interpreted to determine size of particles using Stake’s law. The particles with sizes less than 0.005 mm are classified as clay (C) while particles having sizes between 0.005 mm and 0.075 mm are categorized as silt (M).

PL can be determined using palm rolling method in accordance with ASTM-D4318 [10] as well as fall cone method [48]. In this study, fall cone standard was adopted due to its simplicity and time-efficiency. The cone of apex angle 30° having weight 1.35 N is lowered into soil of varying moisture content under different trials. The plastic limit is termed as the water content at which the penetration of cone is 20 mm in five second of its free fall from a certain height. PL is normally determined using fraction of soil passing from sieve # 40. However, considering the problem at hand, PL was determined using both fraction of soils passing from sieve # 40 (0.425 mm) and sieve # 200 which are referred as PL_40_ and PL_200_ respectively.

## Model development

**Fig 4:**
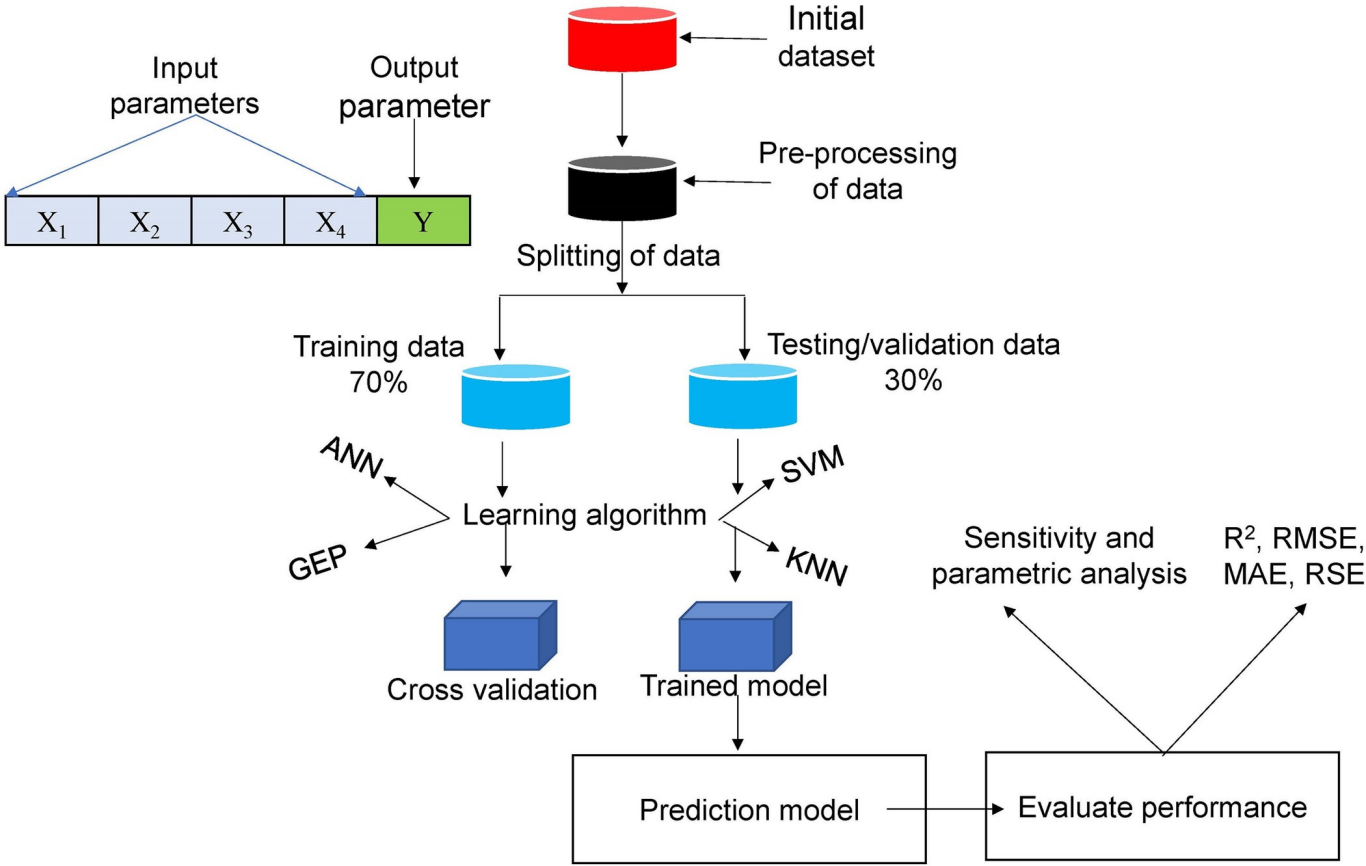
Steps involved in developing prediction model using artificial intelligence techniques.

The processing or compilation of dataset is the first step in developing a prediction model using AI based techniques. The data which is supported by either experimental procedures or in-situ techniques is pre-processed by the selection of suitable and influential input parameters (predictors) in relation to output parameter. Henceforth, splitting of dataset after removing randomness is carried out by dividing it into training and validation categories. The selection of appropriate and robust AI technique is a critical process and requires rigorous knowledge of computer vision. In this study, GEP was selected for the development of prediction model. Afterwards, the model is trained following the principles of programming, and performance is evaluated using different means such as statistical checks and error plots. The working mechanism involved in developing a prediction model is illustrated in Fig 4.

### Dataset compilation

The first step in developing a model involves the selection of appropriate input variable, compiling and processing of data by removing randomness. It is well established that PL is influenced by C, M and S [49]. Therefore, S, M, C and PL_40_ have been considered as the function of PL_200_ as given by Eq 1.


$${PL}_{200}=f(S,C,M,PL_{40})$$ (1)

**Fig 5:**
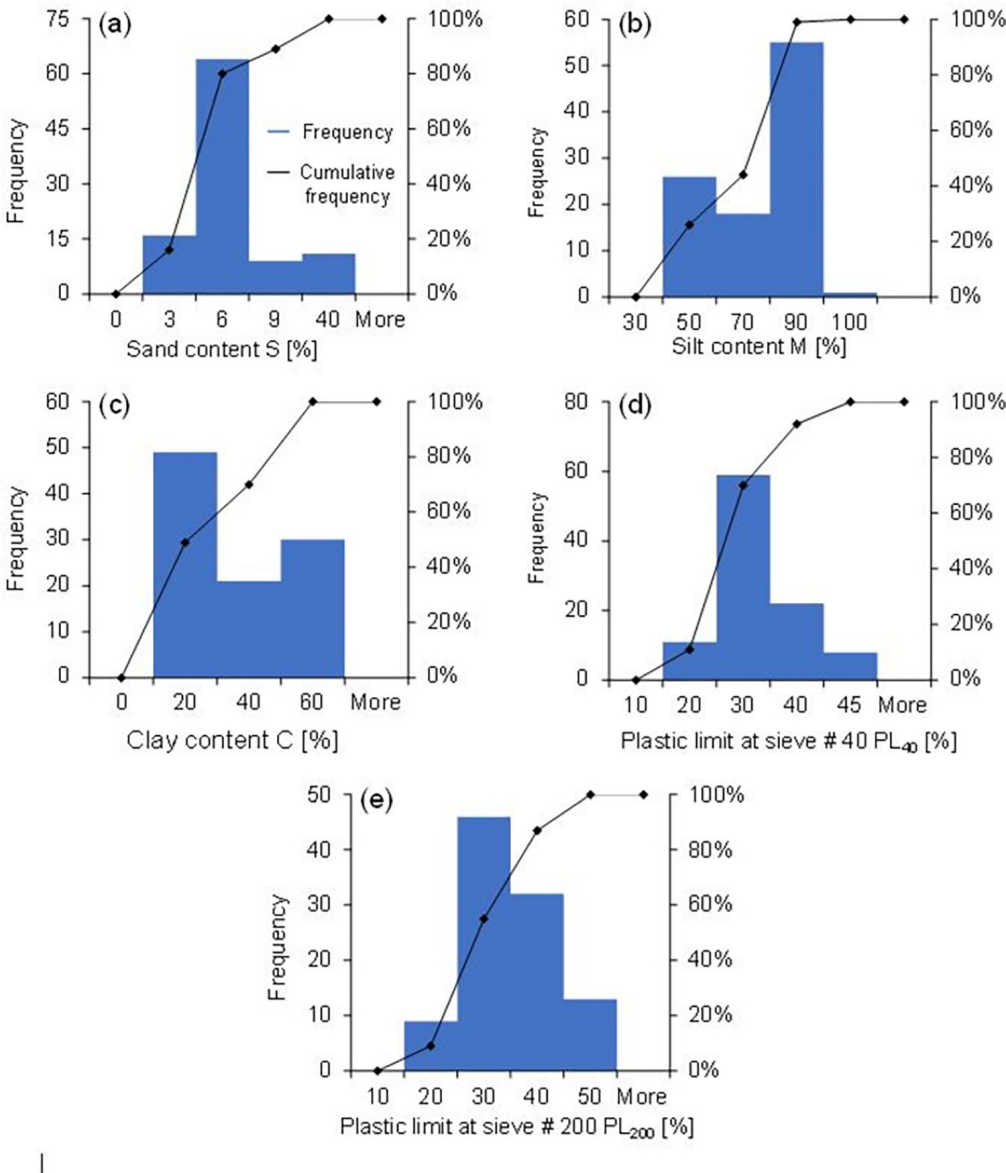
Frequency distribution histograms of experimental data: (a) sand content S [%]; (b) silt content M [%]; (c) clay content C [%]; (d) plastic limit from sieve # 40 passing material PL_40_ [%]; (e) plastic limit from sieve # 200 passing material PL_200_ [%].

As discussed in section 3, the dataset for the modelling purpose was obtained from laboratory testing results. Fig 5 shows the summarized results in the form of histograms of frequency distribution of the data obtained from laboratory experiments. Fig 5(A) shows the results of sieve analysis to obtain sand in the form of the frequency distribution. It was observed that S varies between 2% and 36% and majority of soils have sand content between 3 to 10% indicating the fine grained soils. Fig 5(B) shows results of hydrometer tests in the form of the frequency distribution of silt varying between 34% to 93% with majority of soil samples possess silt between 70% to 90%. Fig 5(C) indicates clay which vary from 5% to 60%. Similarly, Fig 5(D) and 5(E) shows the frequency distribution of PL_40_ and PL_200_, which vary between 14% to 44% and 14% to 54% respectively. This implies that soil samples contain versatility of soil contents and wide range of PL values with low plastic to medium plastic types of soils. Table 1 shows statistical summary of dataset utilized for the development of model in which low standard deviation (SD) values represent less scatter of data around mean average value whereas, higher SD value indicate higher scatter in data.

**Table 1:** Statistics of input and output data for PL_200_ prediction model.

Predictors	Minimum	Maximum	Mean	Std. Deviation
Sand [%]	2	36.2	5.95	4.39
Clay [%]	5	60	27.52	18.6
Silt [%]	34	93	66.45	17.68
Plastic limit, PL_40_ [%]	11	44	27.87	7.29
**Output Data**				
Plastic limit, PL_200_ [%]	23	70	30.97	7.34

### General settings

The accuracy of prediction model using GEP is governed by the selection of appropriate setting of parameters which include as number of genes (N), number of chromosomes and head size [50–52]. Therefore, multiple trials were carried out to choose the best optimal setting of parameters. In this regard, initial selection for the trials was done based on the previous practices adopted by researchers in order to develop prediction models for the evaluation of geotechnical systems. The experimental dataset comprised 100 samples’ properties, was randomly distributed into 70% and 30% for training and validation purpose respectively. The head size, number of chromosomes and genes were selected as 8, 30 and 3 respectively. Table 2 presents the summary of setting of parameters used for developing the GEP based prediction model.

**Table 2:** General setting for prediction models.

General	Model Setting
	PL_200_ [%]
**Genes**	3
**Chromosomes**	30
**Head size**	8
**Set of functions**	+, −, *, ÷
**Linking function**	**+**

### Prediction model evaluation criteria

The evaluation of prediction models is usually performed using a single parameter known as correlation coefficient (R). However, R cannot be solely considered as the reference to evaluate the model’s prediction performance because of its insensitivity to simple mathematical functions such as division and multiplication of output to a fixed value. Therefore, multiple statistical parameters such as root mean square error (RMSE), mean absolute error (MAE) and relatively squared error (RSE) were also considered. The mathematical representation of these statistical parameters is given by Eqs 2 to 5 [53].


$$RMSE=\sqrt{\frac{\sum_{i=1}^{n}{\left(e_{i}-k_{i}\right)}^{2}}{n}}$$ (2)

$$MAE=\frac{\sum_{i=1}^{n}\left(e_{i}-k_{i}\right)}{n}$$ (3)

$$RSE=\frac{\sum_{i=1}^{n}{\left(k_{i}-e_{i}\right)}^{2}}{\sum_{i=1}^{n}{\left(\bar{e}-e_{i}\right)}^{2}}$$ (4)

$$R=\frac{\sum_{i=1}^{n}\left(e_{i}-\bar{e_{i}})(k_{i}-\bar{k_{i}}\right)}{\sqrt{\sum_{i=1}^{n}{\left(e_{i}-\bar{e_{i}}\right)}^{2}\sum_{i=1}^{n}{\left(k_{i}-\bar{k_{i}}\right)}^{2}}}$$ (5)

Where, n is the number of samples, e_i_ is i^th^ experimental output, k_i_ is the i^th^ prediction model response, whereas,$\bar{e_{i}}$ and $\bar{k_{i}}$ are the average values of laboratory and the model responses respectively.

There are several other performance indices that can be deployed to assess the generalization and prediction capabilities of prediction models such as error plots and external validation criteria. The prediction data along with experimental data when lie within ±5 confidence interval is regarded as accurate and reliable [54]. Thus, different kinds of error plots were also utilized to assess the error involved in prediction model.

## Results and discussion

**Fig 6:**
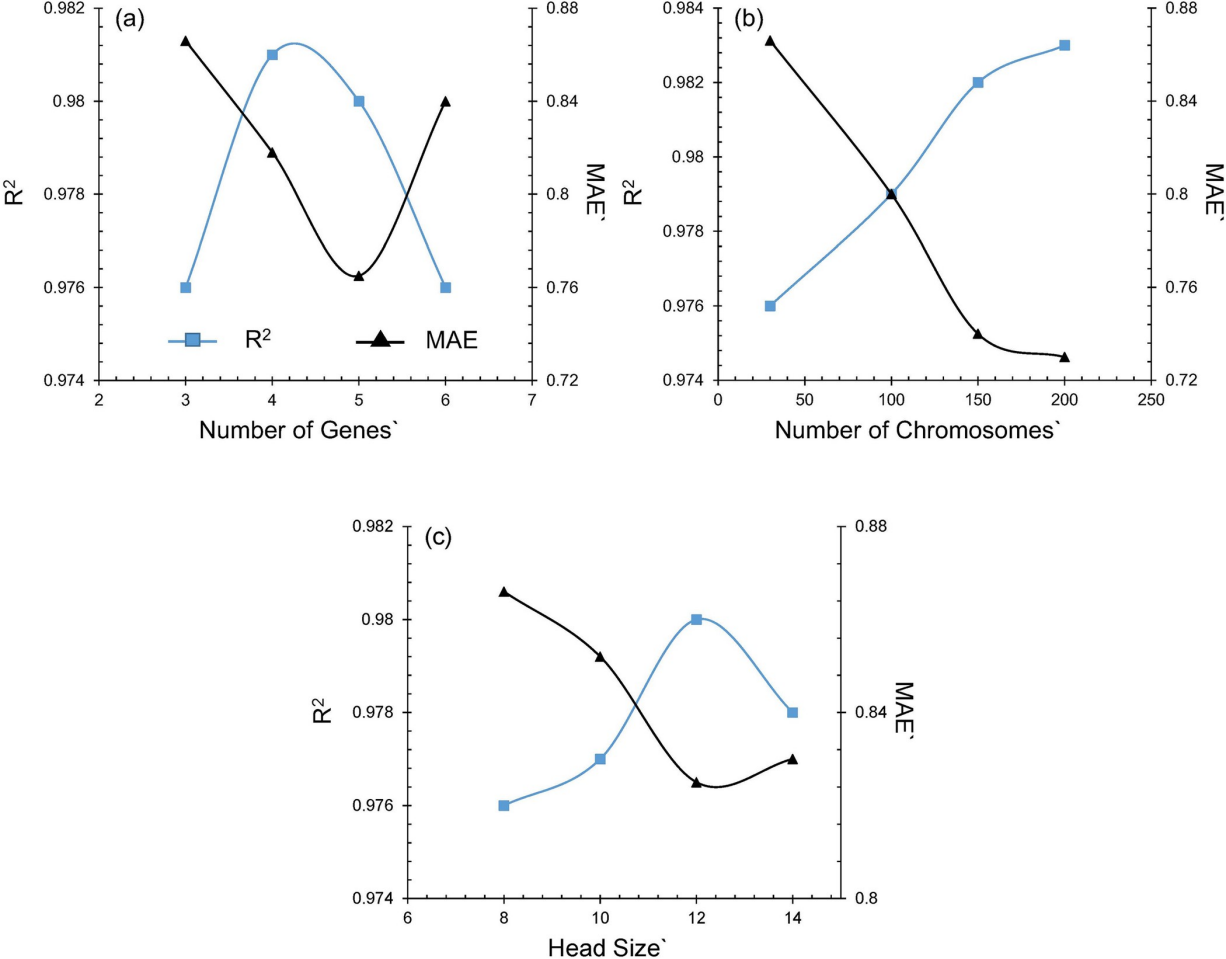
Effect of parametric variation of GEP algorithm on accuracy of predicted plastic liquid: (a) number of genes; (b) number of chromosomes (c) head size.

Fig 6 represents the parametric combination of R and MAE for PL_200_. The study was conducted to determine the optimal setting of three GEP parameters (number of genes, chromosomes and head size) for the prediction of PL_200_. The parametric study was performed by changing one parameter and keeping all other parameters as default. It is evident from the results that R^2^ increases with increase in number of genes, chromosomes and head size up to certain extent and decreases afterwards. This is in agreement with the findings of Oltean and Grosan [55], according to which performance of GEP model increase with the increase in genes up to a threshold point and decreases afterwards due to inability to force complex chromosomes to encode relatively less complex chromosome. Ferrera [29] provided the parameter h_s_ as a measure to determine the complexity and maximum size of parameters involved in developing model. GEP algorithm performs multiple trials of terminals and functions for modelling the parameters inside heads of genes. Therefore, this leads to development of infinite models with varying sizes and shapes. Thus, h_s_ governs the maximum depth (d_max_) and width (b_max_) of Sub-ET in each gene and can be determined using the expressions given by Eqs 6 and 7.


$$b_{max}=[{\left(a_{max}-1\right]}^{*}h_{s})+1]+1$$ (6)

$$d_{max}={\left(\frac{h_{s}+1}{a_{min}}\right)}^{*}\left(\frac{a_{min}}{2}\right)$$ (7)

Where, a_max_ is the maximum arity which is highest number of arguments adopted by the functions whereas a_min_ is the minimum arity (minimum number of arguments adopted by the function) which were taken as 2 and 0 respectively in this study.

Similarly, MAE decreases with increase in number of genes, chromosomes and head size with genes up to 5 and head size 12 while it increases afterwards as shown in Fig 6(A) and 6(C). Thus, default values of setting parameters (genes = 3, chromosomes = 30, head size = 8) were selected as they generate reasonably good accuracy and involve less complexity and time consumption.

**Fig 7:**
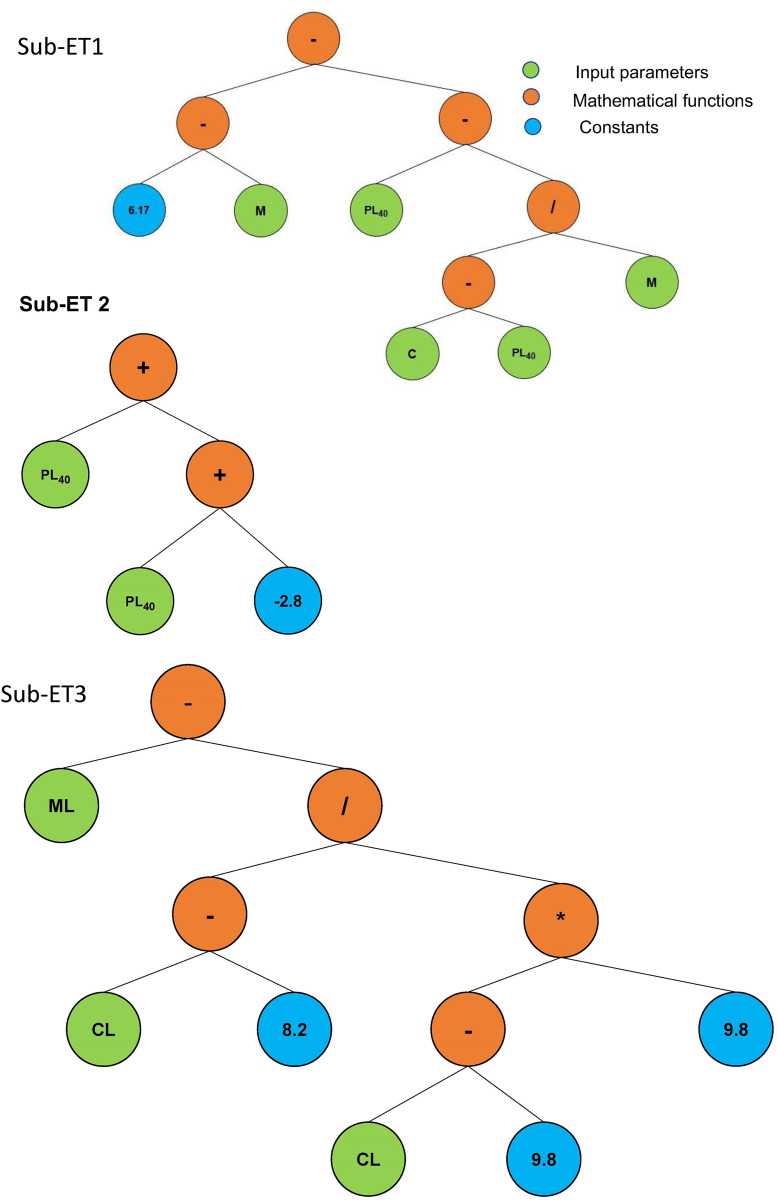
Expression trees [ETs] developed using gene expression programming [GEP].

Fig 7 represents the tree-based structures (ETs) developed using GEP which are further divided into three sub-ETs (Sub-ET 1, sub-ET 2 and sub-ET 3). The principles of Karwa language were followed to derive and decode the simple algebraic expressions from ETs in order to predict PL_200_ as given by Eqs 8 to 11.


$$PL_{200}[\%]=A+B+C$$ (8)

$$A=(6.17-M)-\left[PL_{40}-(\frac{C-PL_{40}}{M})\right]$$ (9)

$$B={PL}_{40}+(PL_{40}-2.84)$$ (10)

$$C=\left[M-(\frac{C-8.2}{(C-9.8)*9.8})\right]$$ (11)

Where, PL_200_ (%) is the plastic limit based on sieve # 200 passing material, A, B and C are the expressions derived from the three ETs and PL_200_ is the summation of A, B and C.

### Performance assessment of model

**Fig 8:**
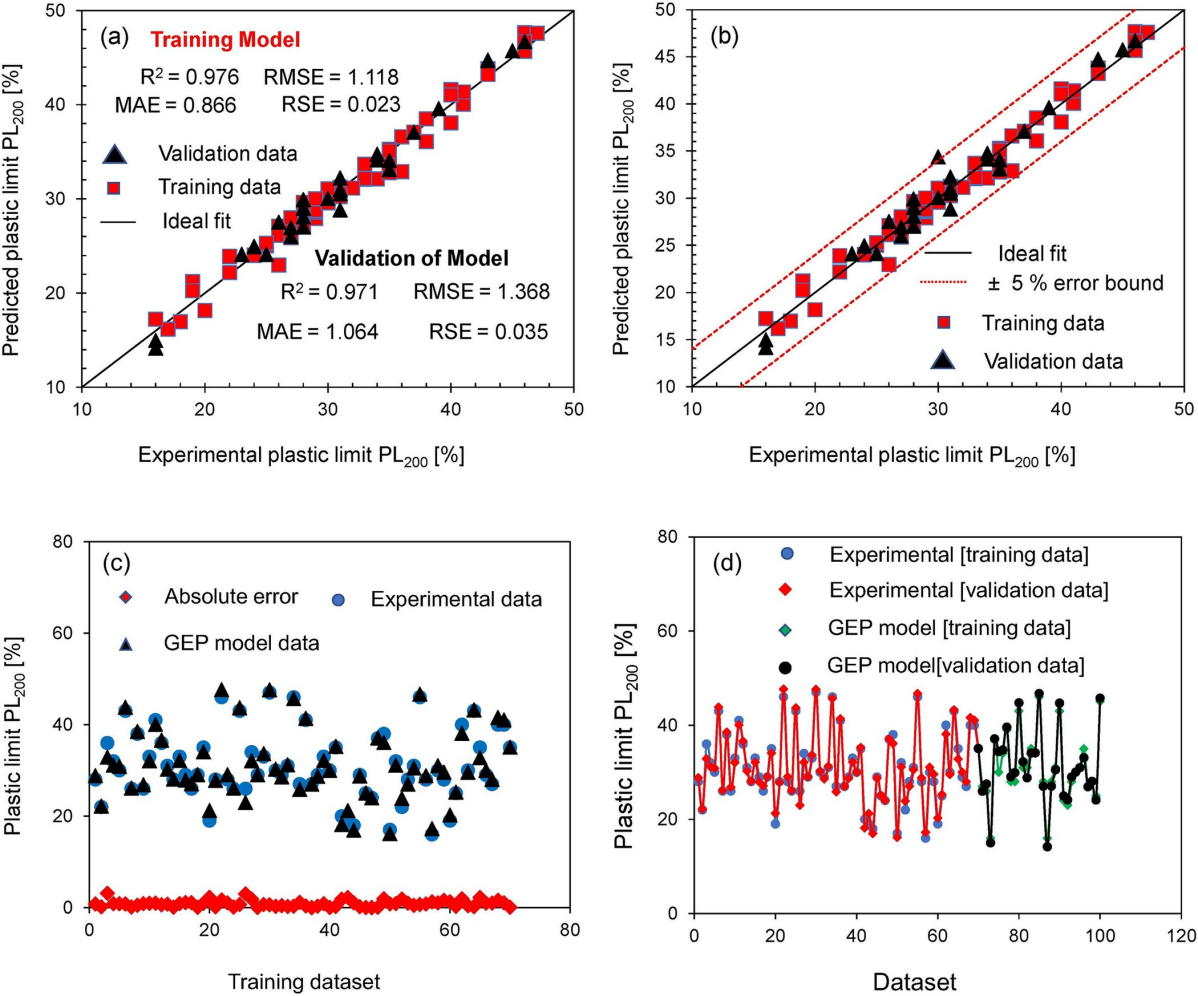
Performance assessment of prediction model based on different criteria; **(a)** comparison of statistical parameters for training and validation data; **(b)** ± 5% error bound for prediction model; **(c)** comparison of experimental data, GEP model data and absolute error; **(d)** comparison of experimental data and GEP prediction model data against training and validation data.

Fig 8 shows the results of performance evaluation of model done by various means Fig 8(A) represents the comparison of different statistical parameters as described in section 4.3, for training and validation datasets. It was found out that the values of R^2^, RMSE, MAE and RSE are 0.976, 1.118, 0.866, 0.023 respectively for training dataset and are 0.971, 1.368, 1.064, 0.035 for validation dataset involved in validating the trained model. According to the literature a prediction model is considered accurate and reliable if it yields values of R^2^ close to 1 and lower values of RMSE, MAE and RSE [50]. This implies that the proposed model has higher prediction accuracy and strong correlation among training and validation data. It is worthwhile to mention that the validation data is used to test the trained model and is not involved in training the model. Thus, it can be regarded as the unseen data and the compliance of trained model to unseen data suggest that the model has been trained effectively and can be employed in field with more confidence.

Fig 8(B**)** shows the plot of error bounds with ±5 confidence interval. The graph was plotted by plotting experimental data (PL_200_) along x-axis whereas prediction responses generated by GEP (PL_200_) along y-axis. A model is deemed accurate if data lies within the pre-defined confidence interval. The results indicate that all the responses lie within ±5 error bounds leading to small error yielded by GEP in relation to input parameters

Fig 8(C) and 8(D) further highlights the error interpretations of the proposed model. Fig 8(C) was plotted between experimental dataset used in training the model and corresponding responses of GEP model along with absolute error. The absolute error is the absolute difference of experimental and prediction data and minimum value of error suggests that model predicts the responses with great accuracy. It is evident from the Fig 8(C) that values of absolute error very less than the mean absolute error in predicting PL_200_.

Similarly, Fig 8(D) draws the comparison of experimental and GEP prediction data against training and validation phases. The findings show that the experimental data and corresponding GEP response data for both cases (training and validation) correlate well and complement one another. The lines of experimental and GEP prediction data overlap each other, and it implies that the error is very less in case of unseen validation data as well. Thus, model’s capability to meet multiple checks and criteria suggest that the model can be used in field with more confidence.

### Sensitivity and parametric study

Sensitivity analysis (SA) is carried out to find out the contribution of individual parameter involved in developing the prediction model. The sensitivity analysis indicates that how sensitive a parameter is in estimating the output. The most sensitive parameter must be dealt with carefully while determining in the laboratory or at the site. The SA can be determined using Eq 12 [39,56]. The value of SA varies between 0 and 100%. The value of zero indicates that the parameter has no significant impact on the model output whereas value close to 100% shows the higher significance and level of sensitivity of parameter.


$$SA=\frac{\sum_{i=1}^{n}\left(h_{i}k_{i}\right)}{\sqrt{\sum_{i=1}^{n}h_{i}^{2}x\sum_{1}^{n}k_{i}^{2}}}$$ (12)

**Fig 9:**
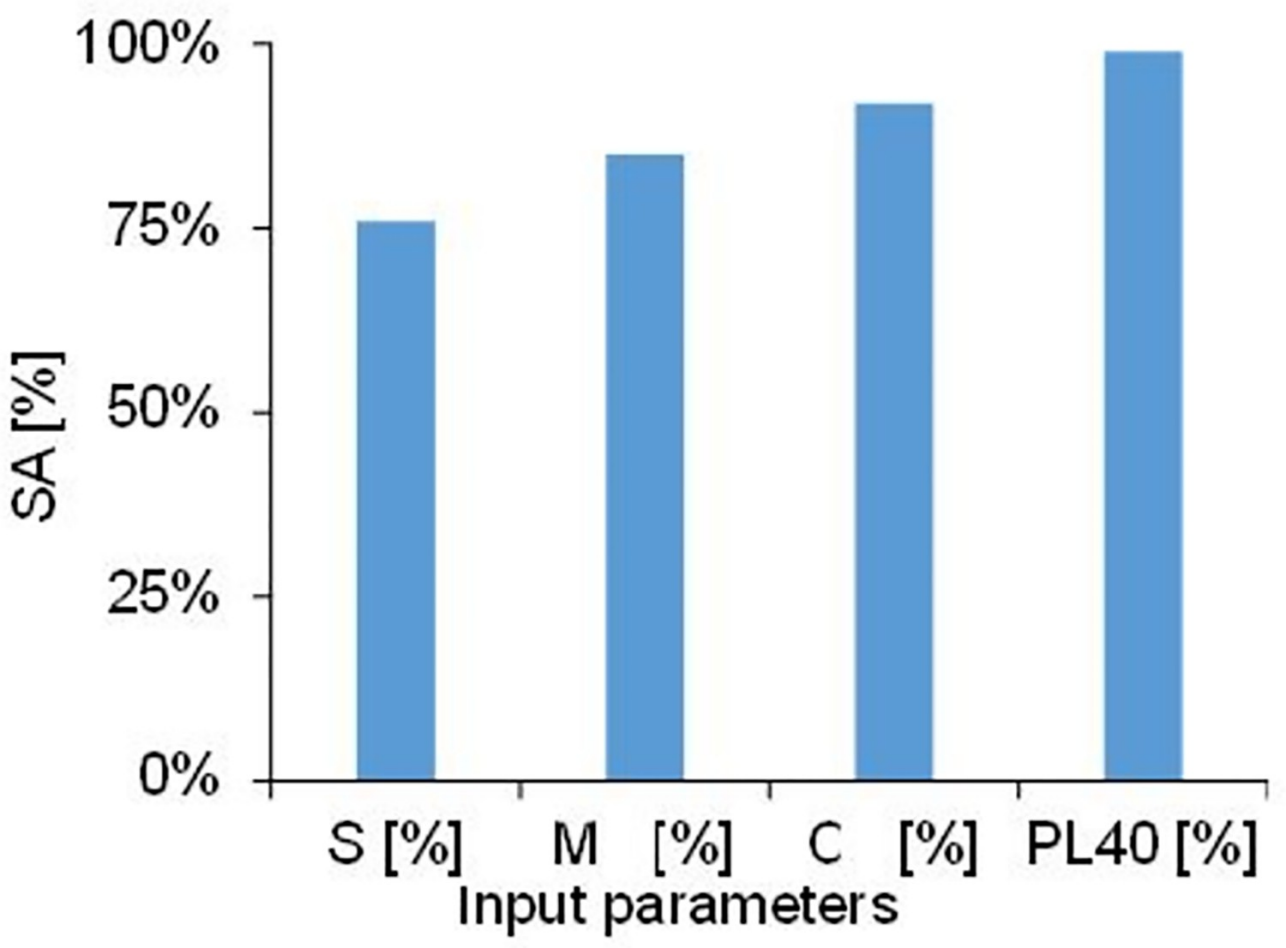
Sensitivity analysis of prediction model based on sensitivity of individual input parameter.

Where, h_i_ is input parameter and k_i_ is the response of predicted model. Fig 9 represents the outcomes of the sensitivity analysis for the proposed prediction model. It was observed that PL_40_ has the most significant impact followed by C, M and S amongst all C is the most critical soil property and S being the least sensitive which is in agreement with the literature. The significance order for all parameters is as PL_40_ > C > M > S. C particles have high surface area than S and therefore can hold more water content and are also regarded as the primary reason of plasticity behavior in cohesive soils.

**Fig 10:**
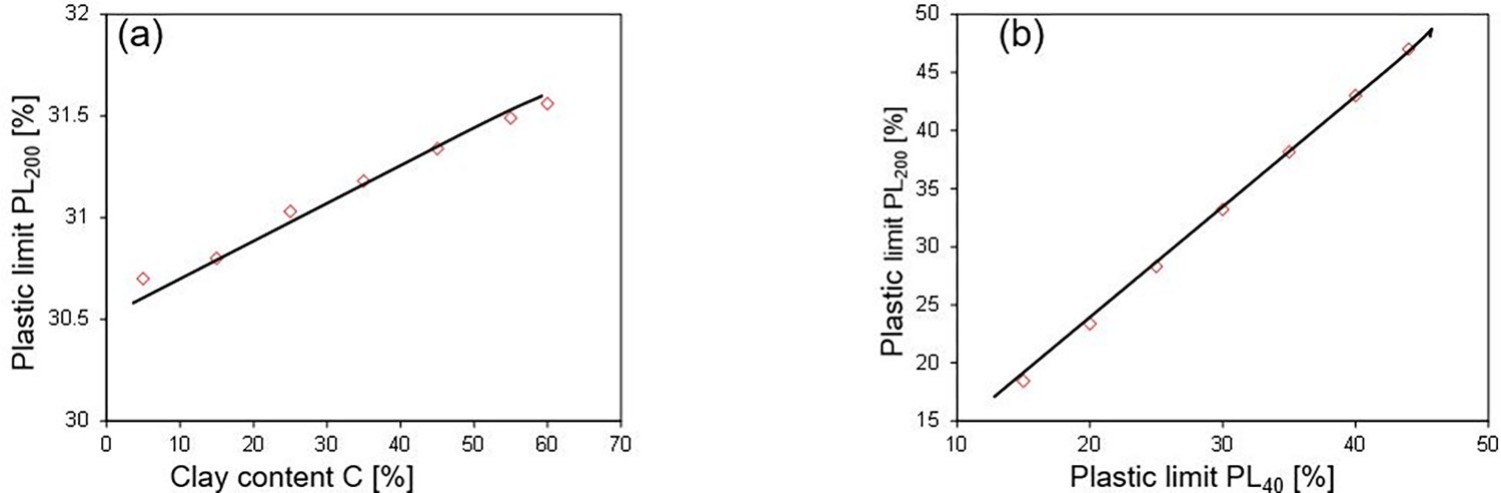
Parametric analysis of input parameters; (a) variation of plastic limit PL_200_ with varying clay content C [%]; (b) variation of plastic limit PL_200_ with varying silt plastic liquid based on sieve # 40 material [%].

In order to justify the fact that correlation model is not mere the correlation but also justifies the physical process, parametric study was also conducted as shown in Fig 10. It is mentioned that the parametric study was only performed on critical parameters determined using SA for the sake of brevity. A parametric analysis is performed by changing one variable around its mean value within the upper and lower bounds of data while keeping all values unchanged at their mean values and then output is regarded. It can be seen that increase in CL causes linear increase in PL_200_, which is because of increase in surface area of soils which leads to enhance the water holding capacity of soils. PL_40_ has the similar trend with PL_200_ and is in line to the findings of Polidori and Lekan.

## Conclusion

Based on soil particles passing through sieve # 200, this study presents a novel prediction model for estimating PL using GEP. The experimental data was utilized to develop the prediction model. The following are the main findings of this research;

- The proposed prediction model incorporates the effect of clay content in order to accurately determine the plasticity behavior of cohesive soils.
- The prediction model was developed using AI based approach i.e., GEP. The model was validated through multiple criteria such as R^2^, RMSE, MAE and RSE. The values of R^2^, RMSE, MAE and RSE against the training data were 0.976, 1.118, 0.866, 0.023 respectively and were 0.971, 1.368, 1.064, 0.035 for testing/validation data.
- The error plot results indicate that the proposed model predicts the responses with the minimal error and responses do not deviate ±5% confidence interval.
- The sensitivity analysis and parametric studies suggest that C is the most critical influencing parameter that can affect PL.
- The proposed model justifies all the criteria of acceptance and can be deployed in field with more confidence.
- The proposed prediction model is applicable to low plastic silty clayey type. Therefore, it is recommended to employ the proposed model to soils having properties ranges within the limits of dataset used in this study. However, future studies may incorporate diverse properties of different types of soils with larger dataset.

## Abbreviations

PL_40_ (%): Plastic limit of soil based on sieve # 40 (0.425 mm) passing material
PL_200_ (%): Plastic limit of soil based on sieve # 200 (0.075 mm) passing material
PL (%): Plastic limit of soil
AI: Artificial intelligence
GEP: Gene expression programming
USCS: Unified Soil Classification System
GP: Genetic programming
S (%): Sand content
M: Silt content
C (%): Clay content
ASTM: American Society for Testing and Materials
GP: Genetic programming
